# Outcomes of totally robotic Roux-en-Y gastric bypass in patients with BMI ≥ 50 kg/m^2^: can the robot level out “traditional” risk factors?

**DOI:** 10.1007/s11701-023-01729-1

**Published:** 2023-10-10

**Authors:** Anne Kauffels, Martin Reichert, Lisa Sauerbier, Annette Hauenschild, Andreas Hecker, Moritz J. Strowitzki, Thilo Sprenger

**Affiliations:** Department of General, Visceral, Thoracic, Transplant and Pediatric Surgery, University Hospital of Giessen, Rudolf- Buchheim-Str. 7, 35392 Giessen, Germany

**Keywords:** Robotic surgery, Roux-en-Y gastric bypass, Super-obesity, Risk factors

## Abstract

Roux-en-Y gastric bypass (RYGB) in patients with body mass index (BMI) ≥ 50 kg/m^2^ is a challenging procedure and BMI ≥ 50 kg/m^2^ has been identified as independent risk factor for postoperative complications and increased morbidity in previous studies. The objective of the present study was to assess whether a BMI ≥ 50 kg/m^2^ and various established risk factors maintain their significance in patients undergoing fully robotic RYGB (rRYGB). A single-center analysis of prospectively collected data of 113 consecutive patients undergoing standardized rRYGB with robotic stapling technique and hand-sewn gastrojejunostomy using the daVinci Xi system. Surgical outcomes were analyzed considering a number of individual perioperative risk factors including BMI ≥ 50 kg/m^2^. The mean BMI of the total cohort was 50.6 ± 5.5 kg/m^2^ and 63.7% of patients had a BMI ≥ 50 kg/m^2^. There were no major surgical and perioperative complications in patients with BMI ≥ 50 kg/m^2^ as well as in those with BMI < 50 kg/m^2^ after rRYGB. We identified female sex and surgeon experience but neither body weight, BMI, metabolic disorders, ASA nor EOSS scores as independent factors for shorter operation times (OT) in multivariate analyses. Complication rates and length of hospital stay (LOS) did not significantly differ between patients with potential risk factors and those without. rRYGB is a safe procedure in both, patients with BMI ≥ 50 kg/m^2^ and with BMI < 50 kg/m^2^. Higher body weight and BMI did affect neither OT nor LOS. A fully robotic approach for RYGB might help to overcome “traditional” risk factors identified in conventional laparoscopic bariatric surgery. However, larger and prospective studies are necessary to confirm these results.

## Introduction

Morbid obesity will remain a challenging health and socio-economic issue with increasing prevalence in many parts of the world [1]. Bariatric surgery is the most effective treatment of morbid obesity in suitable patients. Standardization of surgery as well as surgical specialization and routine use led to a significant reduction of surgical complications and a high level of safety in this field [2, 3].

However, patients with a BMI ≥ 50 kg/m^2^ (formerly designated as “super-obesity”) represent a particularly challenging subgroup and have considerably more often and severe complications and higher mortality rates after bariatric surgery than patients with BMI < 50 kg/m^2^ [4]. Beside technical aspects due to higher amounts of visceral fat, the elevated intraoperative and perioperative complication rates in patients with BMI ≥ 50 kg/m^2^ might be caused by increased frequencies and more serious comorbidities. A vast majority of bariatric surgeons, thus, recommend sleeve gastrectomy (SG) as either single- or two-stage procedure for patients with BMI ≥ 50 kg/m^2^ [5]. However, in a number of patients particularly with gastroesophageal reflux and/or concomitant type 2 diabetes, the Roux-en-Y-gastric bypass (RYGB) might be the more suitable surgical strategy even in patients with BMI ≥ 50 kg/m^2^ (Tables 1, 2).

**Table 1:** Clinicopathological findings of all patients undergoing rRYGB

Feature	Patients with BMI < 50 kg/m^2^ *n* = 41	%*	Patients with BMI ≥ 50 kg/m^2^ *n* = 72	%*
Gender				
Male	11	27	17	24
Female	30	73	55	76
Age (years)				
Mean	44.7		41.53	
SD	10.5		11.5	
Body mass index (kg/m^2^)				
Mean	44.7		53.9	
SD	2.8		3.5	
Body weight (kg)				
Mean	125.8		151.6	
SD	13.4		18.3	
Diabetes mellitus type II				
Yes	21	51	26	36
No	20	49	46	64
Prior abdominal surgery				
Yes	26	63	36	50
No	15	37	36	50
Simultaneous additional surgical procedure(s)				
Yes	14	34	18	25
No	27	66	54	75
Length of hospital stay (days)				
Mean	2.2		2.0	
SD	0.9		0.2	
EOSS (Edmonton Obesity Staging System) Classification				
0	0	0	0	0
1	0	0	0	0
2	37	90	70	97
3	4	10	2	3
4	0	0	0	0
Surgical complications (according Clavien–Dindo)				
0	0	0	0	0
1	0	0	1	1
2	1	2	0	0
3 a	0	0	0	0
b	0	0	0	0
4 a	0	0	0	0
b	0	0	0	0
5	0	0	0	0

*Because of rounding not all percentages might result in 100

**Table 2:** Univariate and multivariate analyses for factors affecting operation time (OT) in rRYGB

Variable	Univariate analysis		Multivariate analysis					
	r^2^	*P *value	Coefficients				t	*P *value
			B		Std. error	95% CI		
Age (years)	0.008	0.3462	–		–	–	–	–
Gender	− 0.065	**0.0065**	− 12.01		3.843	− 19.62 to − 4.39	3.124	**0.0023**
Body weight (kg)	0.004	0.5007	–		–	–	–	–
Height (m)	0.012	0.2416	–		–	–	–	–
Body mass index (kg/m^2^)	0.000	0.8446	–		–	–	–	–
EOSS	0.002	0.6187	–		–	–	–	–
ASA score	0.086	**0.0016**	7.968		4.604	− 1.16 to 17.09	1.731	0.0864
Rank within surgeons learning curve	− 0.325	** < 0.0001**	− 0.445		0.052	− 0.55 to − 0.34	8.658	** < 0.0001**
Simultaneous additional surgical procedure(s) performed	0.072	**0.0042**	20.63		3.676	13.34 to 27.92	5.611	** < 0.0001**

Bold values indicate statistical significance (*P* ≤ 0.05)

*EOSS* Edmonton Obesity Staging System, *ASA* American Society of Anesthesiologists

The robotic platform promises to overcome immanent technical limitations of conventional laparoscopy in bariatric surgery like the counterintuitive movement of instruments and their restricted degree of freedom. Allowing precise intracorporal movements due to articulated instruments, robotics seem to qualify particularly for challenging procedures like RYGB in morbidly obese patients with higher BMI. Nevertheless, although the worldwide use of robotics in bariatric and metabolic surgery is constantly increasing [6], there are still inconsistent data concerning its conclusive role and its economic justification. An evaluation of approximately 80.000 patients who underwent RYGB in 2015 and 2016 recorded within the Metabolic and Bariatric Surgery Accreditation Quality and Improvement Program (MBSAQIP) database showed lower mortality, less bleeding complications, less transfusion requirement, and decreased surgical site infection rates for robotic (rRYGB) compared to conventional laparoscopic RYGB (lRYGB) [7]. However, valid selection criteria for patients who might benefit most from the robotic approach in primary RYGB are still pending. Another recent analysis of the MBSAQIP register data demonstrated higher complication rates and frequently more serious adverse events in patients with BMI ≥ 50 kg/m^2^. Interestingly, in this database analysis, no significant difference in the incidence of serious adverse events was seen in patients with BMI ≥ 50 kg/m^2^ when comparing rRYGB with conventional lRYGB [8].

In the present study, we evaluated established risk factors for RYGB—such as BMI ≥ 50 kg/m^2^, gender, age, and metabolic comorbidities—for intraoperative and postoperative complications, conversions, early postoperative mortality and morbidity as well as operation times (OT) and length of hospital stay (LOS). All patients consecutively underwent fully robotic RYGB (rRYGB) in our institution since establishing the robotic bariatric program. The aim of this study was to identify selection criteria which subgroup of patients might have a particular benefit from rRYGB with regard to perioperative complications and recovery after surgery.

## Patients and methods

Data of all patients consecutively undergoing primary rRYGB at our institution between April 2021 and December 2022 were prospectively collected. All operations were performed by a certified bariatric surgeon. Indications for bariatric surgery were based on multidisciplinary recommendations and according to the national guidelines. The choice of procedure for RYGB was based particularly on comorbidities such as type 2 diabetes and/or gastroesophageal reflux.

All operations were performed with the daVinci Xi system (Intuitive Surgical, Sunnyvale, CA, USA). The surgical technique incorporated a totally robotic approach with robotic stapling technique and hand=sewn two-layer functional end-to-end gastrojejunostomy. Our surgical technique for rRYGB has been described in detail before [9].

A retrospective analysis of the prospectively collected data was performed including patient gender, age, weight, body mass index (BMI), American Society of Anesthesiologists (ASA) classification, Edmonton Obesity Staging System (EOSS), metabolic disorders, and history of prior abdominal surgery. OT (as the continuum from first incision to skin closure, including robot docking and console time), blood loss, intraoperative as well as postoperative complications according to the Clavien–Dindo classification [10] including anastomotic insufficiency, stenosis, re-operation, surgical site infection rates after 30 days, LOS, and readmission rates were assessed.

### Statistical analyses

Statistical analyses were performed using GraphPad Prism (Version 9, GraphPad Software, San Diego, CA, USA). For descriptive statistics, group comparisons of continuous variables were performed either by two-tailed, unpaired Student`s *T *test for two-group comparisons or by one-way ANOVA for global effects and, if applicable, followed by Tukey`s multiple comparison test of each group. Bars in the boxplots depict median, whiskers indicate the minimum to maximum range, the boxes extend from the 25th to 75th percentiles and indicate the interquartile range. Spearman`s rho rank correlation was used for correlation analysis of relevant variables with postoperative length of hospital stay (LOS). Results are given as the Spearman`s rank correlation coefficient (r_sp_) and respective significances.

To determine statistical dependencies between operation time and relevant patient and procedure characteristics, simple linear regression was used for univariate analysis. Variables significantly influencing OT in univariate analysis were included into multivariate analysis by multiple linear regression to determine independent factors that contribute significantly to OT during RYGB.

*P *values ≤ 0.05 indicate statistical significance. Because of the exploratory character of the study, no adjustments of *P *values were performed.

## Results

### Patients’ characteristics and surgical procedures

In total, 113 patients consecutively underwent primary rRYGB. The majority of patients were female (*n* = 85; 75.2%). Mean patient age was 43.7 ± 11.4 years, the preoperative body weight was 142.2 ± 20.6 kg, and mean BMI was 50.6 ± 5.5 kg/m^2^. Forty-seven patients (41.6%) had a manifest and therapy-requiring type 2 diabetes, and 63 patients (55.8%) had prior (open or laparoscopic) abdominal surgery.

Seventy-two patients (63.7%) presented with a preoperative BMI ≥ 50 kg/m^2^ with a mean BMI of 53.9 ± 3.5 kg/m^2^ in this sub-cohort and 41 patients (36.4%) had a BMI < 50 kg/m^2^ with a mean BMI of 44.7 ± 2.8 kg/m^2^_._

A higher BMI was significantly correlated with advanced ASA-Scores (*P* = 0.0078) but not with the presence of type 2 diabetes (*P* = 0.1216) and higher EOSS score (*P* = 0.0809).

In the female cohort (*n* = 85 patients), the mean age was 44.1 ± 10.8 years, the mean preoperative body weight was 137.3 ± 17.5 kg, and the mean BMI was 50.8 ± 5.6 kg/m^2^. Thirty-three female patients (38.8%) had type 2 diabetes and twenty-six (30.6%) had prior abdominal surgery. In the male cohort (*n* = 28 patients), the mean age was was 44.2 ± 10.6 years, the mean preoperative body weight, and BMI were 157.3 ± 22.9 kg and 50.1 ± 5.3 kg/m^2^, respectively. Fourteen male patients (50.0%) suffered from type 2 diabetes and six patients (21.4%) had abdominal surgery in their medical records.

One or more simultaneous additional operative procedure was performed in altogether 32 patients undergoing rRYGB (28.3%). Extended adhesiolysis (either laparoscopically or robotically) had to be carried out in six patients (5.3%). Another six patients (5.3%) underwent simultaneous robotic cholecystectomy due to symptomatic cholecystolithiasis. Fifteen patients (13.3%) had evidence of hiatal hernia, which was closed by posterior hiatoplasty. Two patients (1.8%) had large (> 10 cm) Morgagni hernia, which were closed using mesh repair. Finally, five patients (4.4%) had incidental finding of (later on histologically proven) gastrointestinal stromal tumors (GIST) und underwent partial resection of the remnant stomach.

In summary, additional procedures were performed in 34.1% (*n* = 14) of patients with BMI < 50 kg/m^2^ and in 25.0% (*n* = 18) of patients with BMI ≥ 50 kg/m^2^ (*P* = 0.385).

### Independent contributors to longer operation times (OT) in rRYGB

The mean total OT for rRYGB (including additional procedures in respective cases) was 133.3 ± 24.6 min for all patients ranging between 82 and 207 min. OT significantly decreased over the course of the learning curve. In patients undergoing simultaneous additional surgical procedures (e.g., hiatal hernia repair or cholecystectomy), the OT was 14.5 ± 5.0 min longer which turned out to be significant in both, univariate (*P* = 0.0042) and multivariate analyses (*P* < 0.0001).

**Fig. 1:**
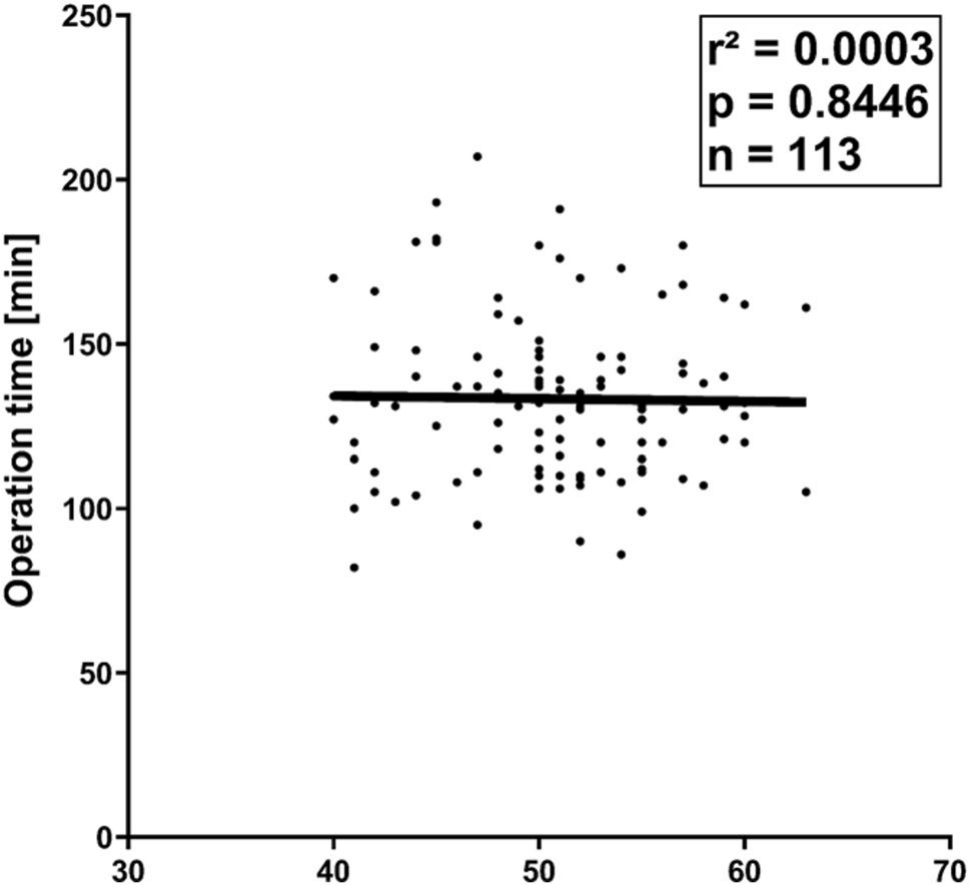
Operation times (OT) in dependence of body mass index (BMI): the patients` BMI did not significant affect OT in rRYGB in this study

Neither the preoperative body weight (*P* = 0.5007) nor the BMI (*P* = 0.8446) had any significant impact on the OT (Fig. 1). Given that the mean BMI of the entire patient cohort was 50.6 kg/m^2^, operation times in patients with BMI ≥ 50 kg/m^2^ (*n* = 72) did not differ from those in patients with BMI < 50 kg/m^2^ (*n* = 41; *P* = 0.3003).

**Fig. 2:**
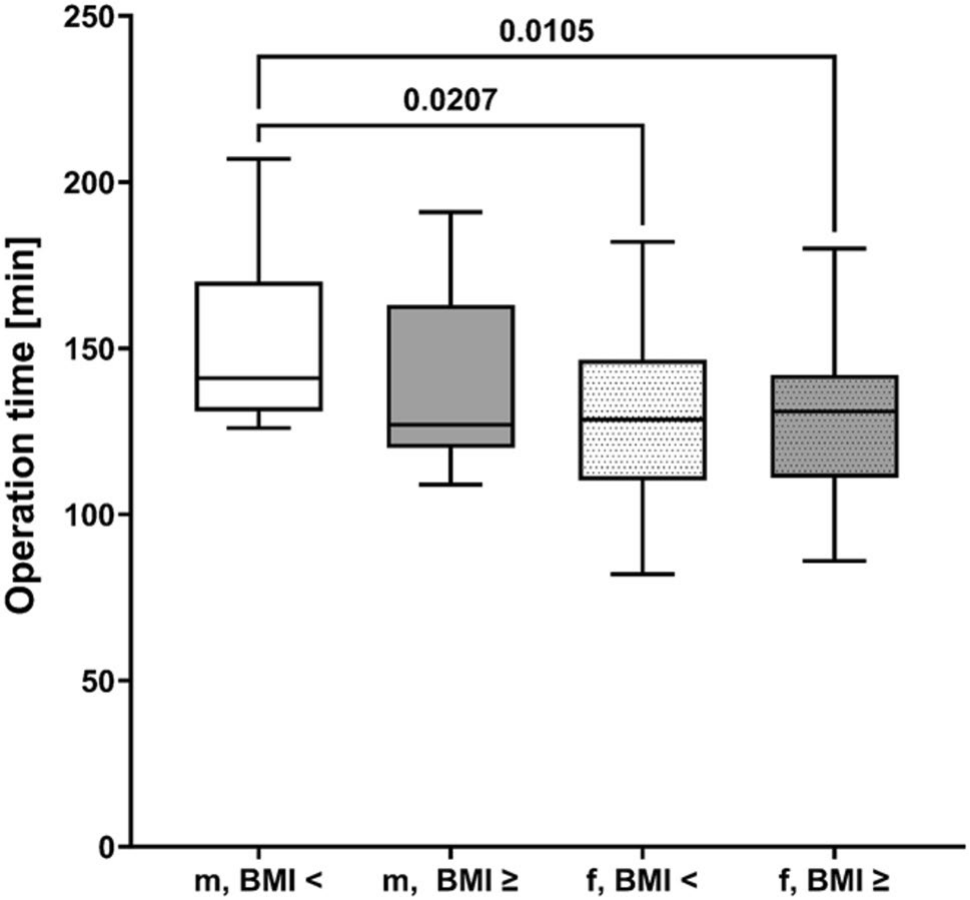
Operation times (OT) in dependence of gender and BMI < and ≥ 50 kg/m^2^: there were significantly longer OT in male patients irrespectively of BMI <  or ≥ 50 kg/m^2^

OT in female patients were significantly shorter compared to those in male patients (*P* = 0.0065). Female sex remained independently correlated with shorter OT in multivariate analyses (*P* = 0.0023). Figure 2 shows the dedicated subgroup analysis of OT regarding female versus male patients with BMI < versus ≥ 50 kg/m^2^. The longest OT were evaluated for male patients irrespectively of BMI.

The strongest independent predictor for shorter OT in our cohort was increased surgeons experience. With increasing operative experience on the robotic platform and overcoming the learning curve, operation times significantly decreased in univariate (*P* < 0.0001) and multivariate analyses (*P* < 0.0001).

The presence of type 2 diabetes (*P* = 0.4306) and a history of previous abdominal surgery (*P* = 0.2173) were not significantly correlated with OT in univariate analysis.

### Correlations with intraoperative blood loss and intraoperative complications

Calculated intraoperative blood loss was minimal (≤ 30 ml) in all patients. There was no significant correlation with patient gender, weight, BMI, presence of diabetes or ASA and EOSS score.

There were no intraoperative surgical complications and no conversions to either laparoscopic or open surgery. One patient had evidence of anaphylactic shock during operation presumably due to perioperative single-shot antibiotics, which required temporarily medical circulatory support as well as antihistaminics and prednisolone therapy, and was, fully recovered, able to be transferred to the regular surgical ward postoperatively.

### Perioperative outcomes and correlations with length of hospital stay (LOS)

In total, two minor complications (1.8%) according to the Clavien–Dindo classification [10] occurred within 30 days after surgery. One patient had postoperative bleeding with intraperitoneal hematoma from a 12 mm assist-trocar site (Clavien–Dindo II) and underwent conservative treatment. One patient undergoing simultaneous cholecystectomy had a surgical site infection after extension of the assist-trocar site access was necessary for removal of the stone-filled gallbladder (Clavien–Dindo I).

There were no major postoperative surgical complications. No anastomotic leakage occurred. No surgical revision or re-operation was necessary. There was no readmission within 30 days. No postoperative intraluminal hemorrhage occurred.

General major perioperative complications (thrombosis, pulmonary embolism, pneumonia) did neither occur within 30 days after operation nor within the further follow-up so far. One patient presented with a self-limiting, presumably drug-induced postoperative thrombopenia after perioperative antibiotic prophylaxis without any clinical consequences.

Due to the limited number of postoperative complications, there was no reasonable correlation with any patient-based risk factors.

The average LOS was 2.1 ± 0.6 days for all patients. Patients with type 2 diabetes had a significantly longer LOS than non-diabetic patients (*P* = 0.044). There was no significant correlation between LOS and gender (*P* = 0.430) as well as BMI (*P* = 0.296) and history of previous abdominal surgery (*P* = 0.237).

## Discussion

Although the evidence-based role of robotics in bariatric surgery is part of an ongoing and controversial discussion, its worldwide use is constantly increasing [11]. Main criticism accumulates in potential higher acquisition and material costs. However, an evaluation of 80,000 patients undergoing RYGB in 2015 and 2016 recorded in the Metabolic and Bariatric Surgery Accreditation Quality and Improvement Program (MBSAQIP) database showed lower mortality, less bleeding complications, transfusion requirement, and surgical site infections for rRYGB compared to lRYGB [7]. Conversely, other studies revealed no significant differences between the laparoscopic and robotic approach and even more complications after rRYGB [12–15].

BMI ≥ 50 kg/m—formerly designated as “super-obesity”—is associated with significantly more and more serious complications after bariatric surgery compared to BMI < 50 kg/m^2^. An evaluation of more than 29,000 patients from the American College of Surgeons National Surgical Quality Improvement Program undergoing all types of bariatric surgery showed higher 30-day mortality rates in patients with BMI ≥ 50 kg/m^2^. This finding particularly affected the RYGB cohort due to significantly more and more severe perioperative and early postoperative complications [4]. Therefore, a vast majority of international bariatric surgeons recommended SG as the procedure of choice for patients with BMI ≥ 50 kg/m^2^ in a representative survey among International Federation of Surgery for Obesity (IFSO) members [5]. Nevertheless, RYGB still has its significance in patients with BMI ≥ 50 kg/m^2^, particularly in those with concomitant gastroesophageal reflux and/or severe metabolic disorders. Moreover, some studies—beside higher remission rates of type 2 diabetes—reported no differences or even better weight loss results after RYGB compared to SG in patients with BMI ≥ 50 kg/m^2^ [16–19].

The primary aim of our study was to evaluate whether a high BMI ≥ 50 kg/m^2^—which is an established risk factor for higher morbidity and mortality after bariatric surgery in general and particularly after RYGB—was likewise associated with adverse outcomes in patients undergoing rRYGB. Notably, in this study, we did not see any significant differences for major intraoperative, perioperative and postoperative surgical complications between patients with BMI ≥ 50 kg/m^2^ compared to < 50 kg/m^2^ as complication rates were considerably low in both patient groups.

Since there were very limited complication rates in both groups of our cohort, the OT might represent a suitable surrogate for individual surgical efforts while the LOS might serve as adequate surrogate for the postoperative recovery of patients. Although patients with BMI ≥ 50 kg/m^2^ suffered from more severe comorbidities in our cohort (occurrence of type 2 diabetes [*P* < 0.0001] and higher ASA score [*P* = 0.0078]), a BMI ≥ 50 kg/m^2^ did neither lead to longer OT nor to longer LOS in this study.

The only published study addressing a comparable issue so far was a MBSAQIP database evaluation [8]. This analysis has been based on 1674 patients with BMI ≥ 50 kg/m^2^ undergoing rRYGB and 24,991 patients with BMI ≥ 50 kg/m^2^ undergoing lRYGB and did not show any differences for postoperative surgical complications between the laparoscopic and robotic approach. However, the study did demonstrate a generally higher incidence of serious adverse events in patients with BMI ≥ 50 kg/m^2^ compared to those with BMI < 50 kg/m^2^ regardless of the surgical approach indicating that a higher BMI represents a valid risk factor for patients undergoing RYGB.

Regarding the OT as an appropriate surrogate for the surgical effort and complexity, we also did not see any significant difference for patients with BMI ≥ 50 kg/m^2^ compared to those with BMI < 50 kg/m^2^. Significantly longer OT were seen in male patients (*P* = 0.0065), within the early phase of the surgeons´ learning curve (*P* < 0.0001), in patients with higher ASA scores (*P* = 0.0016) and when additional surgical procedures were performed (*P* = 0.0042). Except for ASA score, all variables confirmed to be independent factors for longer OT in multivariate analyses.

Male sex was—in contrast to BMI ≥ 50 kg/m^2^—significantly and independently correlated with longer OT. This might be explained by the different distribution of fat tissue with a predominantly higher amount of abdominal fat in male patients. However, even within the male cohort of our study population, there was no significant difference for OT comparing male patients with BMI < 50 kg/m^2^ and BMI ≥ 50 kg/m^2^ (Fig. 2) indicating that the intraoperative challenge in performing rRYGB is not inevitably based on an individually higher BMI. Interestingly, Iranmanesh et al. [20] identified male sex and ASA score > 2 but not BMI ≥ 50 kg/m^2^ as independent risk factors for early postoperative complications after rRYGB in a retrospective analysis of 1276 patients. These factors were equivalent to those we identified for longer individual OT.

Beside higher rates of early postoperative surgical complications, various studies reported frequently higher rates of perioperative (medical) complications, e.g., re-intubation or readmission to ICU and generally higher mortality rates for patients with BMI ≥ 50 kg/m^2^ undergoing bariatric surgery [21, 22]. For rRYGB, fast recovery with a mean LOS of 2.12 days and no significant differences in LOS between patients with BMI < 50 kg/m^2^ and BMI ≥ 50 kg/m^2^ in our study might have led to frequently limited rates of perioperative (non-surgical) complications, such as deep vein thrombosis, pulmonary thromboembolism or pulmonary exhaustion in both groups.

Our study has a number of limitations. With its retrospective nature, it was neither designed to compare outcomes of patients undergoing rRYGB and lRYGB in a randomized controlled fashion nor to match our patient cohort with a comparative collective of patients treated with conventional lRYGB. References to established operative and perioperative risk factors for patients undergoing lRYGB—such as BMI ≥ 50 kg/m^2^— can, therefore, only be interpreted with caution. However, our study reveals the necessity for prospective controlled multicenter trials to clarify the role of robotics in bariatric surgery and its capability to reduce operative and perioperative complications in patient subgroups with particular risk factors like BMI ≥ 50 kg/m^2^.

## Conclusion

rRYGB is a save procedure and can be carried out with very low intraoperative and perioperative complication rates in patients with BMI ≥ 50 kg/m^2^ and with BMI < 50 kg/m^2^. A higher BMI was neither identified as risk factor for complications, higher morbidity or mortality nor was it associated with increased OT and LOS in patients undergoing rRYGB. Male sex and surgeon experience were the only independent factors influencing OT but not perioperative complications, morbidity, and mortality after rRYGB.
